# Affective touch experiences across the lifespan: Development of the Tactile Biography questionnaire and the mediating role of attachment style

**DOI:** 10.1371/journal.pone.0241041

**Published:** 2020-10-28

**Authors:** Mercedes I. Beltrán, H. Chris Dijkerman, Anouk Keizer

**Affiliations:** Department of Experimental Psychology, Helmholtz Institute, Utrecht University, Utrecht, The Netherlands; Universita degli Studi di Udine, ITALY

## Abstract

The sense of touch develops early in life and becomes a determinant aspect of our personal narratives, providing crucial information about the world around us and playing a prominent role in affective and social interactions. In this study we aimed to explore whether individual differences in touch experiences across the lifespan are related to adult attachment styles and to perceived touch deprivation. For this we first developed an instrument, namely the Tactile Biography, to quantify individual differences in affective touch experiences throughout life. Secondly, we performed a set of regressions models and a mediation analysis to investigate the role of attachment in relation to both the tactile history and perceived touch deprivation. We found that experiences of affective touch during childhood and adolescence seem to be closely associated with adult attachment styles and adult social touch experiences. Avoidant attachment appears to serve as a mediator in the relationship between earlier (childhood/adolescent) and later (adult) affective touch experiences, as well as between earlier affective touch experiences and perceived touch deprivation. These findings offer further support to existing literature, providing novel insights for the fields of social affective touch and attachment research.

## Introduction

The ontogeny of the sense of touch takes place in the early stages of the intrauterine life [1]. The first sensations that are experienced are thought to be tactile sensations, occurring somewhere between the 4 to the 7 week of gestation [2]. From there on, the sense of touch further develops and becomes a determinant aspect of our personal narratives. On a daily basis we interact with the world and others around us and touch can have a central role in these interactions. Throughout the lifespan, the sense of touch provides not only crucial information about the physical world, but also plays a prominent role in affective and social interactions (see [3] for a full review). As a natural part of our relationship with others, interpersonal touch can constitute an essential part of our life, our memories and personal stories.

Interpersonal affective touch is thought to be supported by a C-tactile (CT) afferent fibers system which is optimally stimulated by gentle caressing velocities and at human skin temperature [4, 5]. This type of touch is key for the communication of emotions and for social affiliative behaviors [6, 7]. Additionally, affective touch has been associated, among others, with psychological well-being [8], body representations [9] and buffering of pain and stress systems [10, 11].

The way we perceive, process and react to interpersonal touch can be affected by multiple contextual factors [12]. Pleasantness ratings of touch have been shown to be shaped by information coming from other senses [13, 14], socio-cognitive abilities [15] and cognition and linguistics [16]. Other aspects stemming for individual differences related to the touch experiences themselves, such as attitudes towards touch [17] and levels of touch exposure have been also described as factors affecting touch perception. Sailer and Ackerley investigated affective touch perception in a group of individuals with self-reported low touch exposure [18]. Contrarily to expectations, they found that low touch exposure was associated with a less pleasant perception of touch, in particular when it was given at caressing velocities. Consequently, individuals that perceive themselves as lacking affective touch experiences, and consider themselves touch deprived, offer an important clue about how the personal history of touch can influence perception.

Our experiences of touch across the lifespan may also affect how we experience and perceive affective touch later in life. In this sense, the particular occurrence of interpersonal touch experiences that are valued as negative could influence the way we later perceive and react to affective tactile stimuli. Few studies have focused on this last issue. Research has previously shown that childhood trauma, in terms of emotional or physical abuse, is related to chronic pain [19, 20]. In particular, laboratory research on pain perception has suggested that traumatic experiences in childhood are associated in particular with the affective, but not sensory, components of pain [21]. Though of paramount importance, the specific association between interpersonal touch experiences valued as negative and affective touch, still needs to be addressed.

All in all, individual differences in the personal tactile affective history, could modulate how we perceive and react to affective touch. Perhaps, these differences could also exert a regulatory effect on the neural correlates and physiology behind the tactile experience.

Attachment style can be understood as a systematic pattern of relational expectations, emotions, and behaviour that results from an individual’s history of attachment experiences [22]. Since early childhood, stemming from experiences with primary caregivers, inner working models of self and others are developed influencing perceptions throughout the life span [23]. As a result of these models, and depending as well on the quality of current interactions, individuals can exhibit a more secure style of attachment or varying levels of anxiety and avoidance in interpersonal relationships [24]. There is a level of consensus among authors that attachment related anxiety and attachment related avoidance, represent dimensions of the attachment spectrum. Variations along these dimensions can help to understand individual differences in attachment [24, 25]. These constitute a source of differences not only in the establishment and quality of interpersonal relationships but also in the availability and use of affect and stress-regulation strategies [26, 27]. Understanding factors that contribute to modulate the development of attachment processes is crucial. For example, suboptimal bonding in at risk infants has been linked to impaired epigenetic, hormonal and neuronal development [28] and in adults, attachment style has been related to emotion regulation strategies and development of psychopathology [29, 30].

The specific role that affective touch has in the facilitation attachment has been put forward by a number of studies. During early life, parent-infant day-to-day physical contact has been considered a pivotal factor in the development of attachment security [23, 31, 32]. Both Ainsworth [31] and Anisfeld [32] showed that closer and more frequent physical contact between mothers and their young infants promoted the development of a more secure attachment. From the earliest encounters after birth touch holds a communicative and regulatory function [33]. Mercuri et al. [34] showed that in the postpartum both parents interact with their newborns by means of diverse touching behaviours, with a preference for stroking and caressing. This and other studies reported the presence of affective maternal touch after exposure to a stressful situation for the mother and her infant (Still Face paradigm). Their findings underscore the relevance of affective touch for the development of regulatory strategies in the infant [35–38]. Moreover, higher frequency of affective touch in infancy was found to predict cognitive and neurobehavioral development in very early infancy [39, 40] and the development of connectivity in areas related to the social brain across the first 10 years of life [41]. The influence of early parental touch on adult attachment styles has been less investigated. A previous study by Takeuchi and colleagues explored self-reported frequencies of early parental touch in relation to attachment-related mental representations of ‘self’ and ‘others’ [42]. According to their results, a poorer ‘other’ image, which would be potentially indicative of higher avoidance, was predicted by lower self-reported frequencies of early parental touch.

Later in life, the interpersonal touch and social interactions are also associated. Touch in close relationships was found to promote attachment security [43] and relational and psychological well-being [44]. Regarding attitudes to touch, previous research indicates that individuals exhibiting an avoidant attachment show aversive attitudes towards social touch [45] and that anxiously attached individuals tend to use and seek more affective touch in close relationships [46]. Krahé et al. investigated whether attachment style modulated the effects of affective touch on reduction of pain, finding that anxiously attached individuals show enhanced positive effects of affective touch for pain reduction [47]. The same authors later showed in an experimental setting that attachment anxiety affected discrimination in self-reported pleasantness between affective and non-affective touch, with increasing levels of attachment anxiety leading to a decrease discrimination between conditions [48]. In a recent study Spitoni et al. [49] analysed whether the perception of affective touch differed between adults with different attachment patterns. In contrast with adults with an organized attachment pattern, those with disorganized attachment did not perceive affective touch as pleasant, showing preference for non-affective touch.

In the current study we aimed to explore whether/how individual differences in affective touch experiences across the lifespan, including the presence of negatively valued experiences, are related to individual differences in adult attachment styles and to perceived touch deprivation. For this, we first developed an initial version of the Tactile Biography, a questionnaire to quantify individual differences in affective touch experiences throughout life. Secondly, we performed a set of regressions models and a mediation analysis to investigate the role of attachment in relation to both the tactile history and the perceived touch deprivation. Considering previous research highlighting the importance of early tactile interaction for the development of secure attachment [31, 32, 50], we hypothesised that a greater frequency and satisfaction with tactile affective experiences across the lifespan would be reflected in a more secure attachment organization. Consequently, we expected that a more secure attachment, as showed by lower levels of attachment-related anxiety and avoidance would echo in lower reported levels of perceived touch deprivation.

## Materials and methods

### Participants

229 adults aged between 18 and 81 years of age (M = 37.52; SD = 13.74) participated in this study. Recruitment was performed internationally via social media and locally with university postings at Utrecht University, The Netherlands. Participants responded to a set of demographic questions together with four questionnaires that were hosted online using the Gorilla platform [www.gorilla.sc] [51]. Questionnaires were offered in English, Dutch and Spanish. Exclusion criteria were insufficient comprehension of one of these languages and a self-reported history of a psychiatric or neurological diagnosis. Student participants from Utrecht University could receive student credits as a compensation for participation. The study was performed according to the Declaration of Helsinki and was granted ethical approval by the ethics committee of the Faculty of Behavioural Sciences, Utrecht University (protocol number FETC19-017). All participants gave informed consent prior to the start of the experiment.

### Questionnaires

Participants filled in an initial version of the Tactile Biography, a self-designed instrument to quantify and describe affective touch experiences across the lifespan. A set of questions on demographic data was also completed together with three validated questionnaires in order to assess adult attachment style, touch deprivation and experiences and attitudes towards positive touch.

#### Tactile Biography

Participants were presented with an initial version of the instrument containing a set of 37 questions referring to experiences of affective touch in close relationships throughout their personal history. With the intention of registering the complexity of affective touch experiences, the instrument was designed to included questions to be responded on a Likert scale, yes/no questions, multiple choice questions and open questions. The diversity of items reflects the attempt to combine both quantitative and qualitative information over the personal stories of touch. Affective touch experiences were defined for the questionnaire as activities where people touch each other showing positive affection, such as caressing, hugging, kissing, holding hands, etc. A response could be selected for each question according to the highlighted indication on top of each section of the questionnaire.

Thirty-four (34) items were presented as statements to be answered in a 5-point Likert scale: for 14 items the 5-point scale ranged from *never* to *very frequently* where the participant could indicate how frequent the stated event occurred (5 of these items were follow-up questions only presented if the participant responded positively about whether they had or have had a romantic relationship—2 items—or had children—3 items -); in 17 items participants were presented with a 5-point scale which extremes were labelled as *not at all true* and *extremely true* selecting a response to indicate the degree to which each statement applied to them (6 of these items were nested in the same question); 3 items (also nested in the same question) inquired about satisfaction with the amount of affective touch experienced in different life moments and the extreme values of the 5-point scale were labelled as *not at all happy* and *extremely happy*. All scores were computed so that greater scores would reflect higher frequency/satisfaction with touch.

Additionally, three other items were presented. One item was presented in multiple choice layout in which the participants could identify from a range of emotions (5 with a positive value, 5 with a negative value) those generated by affective touch in close interpersonal relationships. Selecting more than one option was possible and an extra open option was offered to register emotions that were not listed. Another item was also presented in multiple choice layout in which the participant could indicate an historic preference to give or receive affective touch in close relationships (selecting both of neither was also possible). Finally, a yes/no question was presented in which the participants could indicate whether they recognized in their personal history the presence of negative or unpleasant experiences involving interpersonal touch. Participants were given the option to leave the question unanswered and to indicate shortly the type of experience motivating the answer.

To ascertain component structure of this questionnaire, a principal component analysis was performed and is described in the results section of this study. Mean scores of the items corresponding to each of the identified components were further used for data analysis.

#### Experiences in close relationships—Revised

Participants completed the Experiences in Close Relationships—Revised questionnaire [52]. The ECR-R is well-validated instrument to measure individual differences in adult attachment style [53]. This 36-item self-report questionnaire assesses adult attachment style yielding scores in two dimensions: attachment-related anxiety and attachment-related avoidance. Items are presented with a 7-point scale from *strongly disagree* to *strongly agree* and are averaged per dimension, with higher scores denoting greater attachment-related anxiety or avoidance. All items were formulated to account for attachment in close relationships in general and were randomly presented as suggested by the authors of the instrument. In the present sample, the ECR-R showed excellent internal consistency, with Cronbach’s α values of 0.91 for attachment-related anxiety and of 0.86 for attachment-related avoidance. In accordance to research supporting a non-categorical conceptualization of attachment, dimensional scores of the ECR-R were further treated as continuous variables [54].

#### Touch Deprivation Scale

The Touch Deprivation Scale is a self-report questionnaire validated in a student sample [55]. This questionnaire consists in 14 items in which the participants is presented with statements about the frequency of touch in their current life and their current desire to be touched. All items can be responded in a 5-point Likert scale ranging from *strongly disagree* to *strongly agree* and are coded to indicate higher perceptions of touch deprivation. Items are grouped in three different components: ‘absence of touch’, ‘longing for touch’ and ‘use of sexual behaviour to be touched’ and mean scores for each subscale are calculated. After reliability analysis scores corresponding only to two subscales were used in the present study: absence of touch (Cronbach’s α = .82) and longing for touch (α = .71). Reliability score of ‘use of sexual behaviour to be touched’ was considered poor (α = .55) and the component was excluded from further analyses.

#### Touch Experiences and Attitudes Questionnaire

The Touch Experiences and Attitudes Questionnaire (TEAQ) consists of a validated 57-item self-report measure to determine attitudes toward and experiences of positive touch [56]. The TEAQ presents a six-component structure: *a*. interpersonal physical touch experiences and attitudes with friends and family (Friends and Family Touch, FFT), *b*. current experiences of intimate touch in emotionally close relationships (Current Intimate Touch, CIT), *c*. positive touch experiences during childhood (Childhood Touch, ChT), *d*. fondness for positive self-care behaviours, such as skin care and grooming (Attitude to Self-Care, ASC), *e*. attitudes towards intimate touch experiences in emotionally close relationships (Attitudes to Intimate Touch, AIT) and *f*. level of comfort with physical touch received from people the individual is not emotionally close to (Attitude to Unfamiliar Touch, AUT).

All items of the questionnaire can be responded in a 5-point Likert scale ranging from *strongly disagree* to *strongly agree* and average scores are calculated for each component.

In the present sample Cronbach’s α examination suggested good to high reliabilities of the TEAQ subscales: FFT (α = .90), CIT (α = .83), ChT (α = .89), ASC (α = .74), AIT (α = .89) and AUT (α = .71).

### Data analysis

Data were analysed using SPSS Version 23.0 (IBM Corp., Armonk, N.Y., USA). Standardized values (z scores) and Mahalanobis distances were used to identified univariate and multivariate outliers respectively for the questionnaires data. Normality was assumed due to the Central Limit Theorem.

#### Principal component analysis

To test the component structure of the Tactile Biography (TBIO), a principal component analysis (PCA) with direct oblimin rotation was carried out on the raw data of the questionnaire, using only the 34 items that were measured on a 5-point Likert scale. Prior to the analysis the scores of the responses for negatively phrased items were reversed.

Kaiser- Meyer-Olkin (KMO) value and measures of sampling adequacy (MSA) of individual variables together with Bartlett’s test of sphericity were inspected to asses appropriateness for the PCA analysis [57]. Missing values were excluded pairwise and an item correlation matrix was obtained. The number of components retained was determined by eigenvalues over Kaiser’s criterion of 1 [58], and screening of the Cattell’s screeplot [59]. Items loading on more than one component were considered poor and consequently removed, as well as items with communalities < 0.3. Item loadings were inspected in the pattern matrix removing items with loadings below 0.40.

After identification of components, reliability analysis was performed and if items which deletion would significantly decrease Cronbach’s α were identified, these were also removed. Further psychometric properties were explored by examining correlations between the TBIO components and the subscales of the TEAQ questionnaire.

#### Regression and mediation

To test specific associations between the questionnaire data, a set of univariate and multivariate regressions was conducted between subscales of the ECR-R and TDS and the 4 components identified for the Tactile Biography via the principal component analysis. Regression models were performed adjusting age and country of origin to address potential confounding effects of these variables.

Upon significant results of the regression and to test a potential mediator role of attachment, questionnaire data were included in a mediation analysis using the PROCESS macro for SPSS developed by Hayes [60]. Indirect effects of *childhood/adolescent touch experiences* on *adult touch* and *perceived touch deprivation* through *individual differences in attachment* were explored. Considering the results of the initial regression models, mediation was only performed when significant results were found for regressions: (1) between the outcome and the predictor, (2) between the outcome and the mediator (3) between the mediator and the predictor (see [61] for guidelines). Mediation models were built separately for each outcome variable. In our mediation analyses, adult touch experiences and touch deprivation served as the dependent variables, attachment style was explored as mediator, and childhood/adolescent touch experience was the independent variable. Standardised indirect effects are reported with 95% bootstrapped confidence intervals from 5000 re-samples [62].

Additional analyses were performed for other items of the Tactile Biography that were not included in the component scores. In those cases, the statistical test used is reported in the result section.

## Results

Full demographic characteristics of the study sample are presented in Table 1.

**Table 1:** Sample characteristics.

	N = 229
Age [Mean ± SD]	M = 37.52 [18–81]; SD = 13.74
Gender [%]	81.2% Female
	17.0% Male
	.9% Non-binary/Gender variant
	.9% NA
Country of origin [%]	60.7% Argentina
	33.2% Netherlands
	6.1% Other countries
Instruction level [%]	8.3% High school
	23.1% Vocational training
	33.2% University of applied sciences
	35.4% University graduate
Family situation [%]	17.9% living alone
	59.4% living with partner and/or children
	9.2% with partner and/or children (not living together)
	13.5% living with parents/housemates
Having children*[%]	49.3% Yes
	50.7% No
Attachment-related anxiety (ECR-R)	M = 3.26 SD = 1.16
Attachment-related avoidance (ECR-R)	M = 3.39 SD = .86
Absence of Touch (TDS)	M = 2.19 SD = .75
Longing for Touch (TDS)	M = 2.07 SD = .89
Adult Touch Experience (TBIO)	M = 4.00 SD = .73
Child/Adolescence Touch Experience (TBIO)	M = 3.67 SD = .92
Comfort with Touch (TBIO)	M = 4.25 SD = .72
Fondness of Touch (TBIO)	M = 3.91 SD = .80
Self-reported negative experience related to interpersonal touch* [%]	34.1% Yes
	60.3% No
	5.7% NA
Affective touch preference*[%]	Active (give touch) 15.3%
	Passive (receive touch) 13.1%
	Both 70.3%
	Neither 1.3%

Sample characteristics and mean values from questionnaires. Names of subscales are followed by indication of the main questionnaire to which they belong. ECR-R = Experiences in Close Relationships -Revised. TDS = Touch Deprivation Scale. TBIO = Tactile Biography. Data marked with * proceeds from items included in Tactile Biography.

### Tactile Biography

#### Principal Component Analysis (PCA)—Tactile Biography

Confirmation of sample adequacy for the analyses was attained with a significant Bartlett’s test at p < 0.001, acceptable values for Kaiser- Meyer-Olkin measure, KMO = .878, and inspection of the anti-image correlation matrix that rendered MSA ≥ .682 for individual variables [57].

PCA was then performed sequentially eliminating poor items (as described in Data Analysis section). Based on Kaiser’s criterion in conjunction with examination of the scree plot a five-component structure was initially retained, on basis of 32 items, and accounting for 61.6% of the variance. One component grouped 4 items consisting in follow-up questions in which several participants had no data. As this component was difficult to interpret due to disparity of items and missing data, was considered as a *follow-up questions component* and we decided to exclude it from further analysis. The structure of the resting components was re-confirmed when running a new PCA removing the aforementioned items. This final analysis yielded a 4-component structure, based on 28 items, that accounted for a 60.95% of variance. Complete detail of component items and loadings is presented in Table 2. Internal consistency of the components was explored by means of reliability analysis yielding good to excellent values of Cronbach’s α (range 0.73–0.94).

**Table 2:** Item loadings Tactile Biography.

Tactile Biography Items	Components			
	1 (36.25%)	2 (12.95%)	3 (6.01)	4 (5.74)
1. How frequently you experienced affective touch in different life moments? (Childhood).	0.949			
2. How happy you are with the amount of affective touch you experienced in close relationships, in different moments of your life? (Childhood).	0.934			
3. As a child I received affective touch from family members (parents/caregivers).	0.920			
4. As a child my parents/caregivers would use bodily contact (e.g.: caressing, hugging, etc) to comfort me when ill/distressed.	0.897			
5. As a child my parents/caregivers would use bodily contact (e.g.: caressing, hugging, etc) to congratulate me/ give me positive feedback.	0.813			
6. I am satisfied with the amount of affective touch I received throughout my personal story.	0.694			
7. While growing up, upon stressful situations I would go to my parents/caregivers in search of affective touch (hugs, cuddling, caressing)	0.689			
8. How happy you are with the amount of affective touch you experienced in close relationships, in different moments of your life? (Adolescence).	0.607			
9. How frequently you experienced affective touch in different life moments? (Adolescence).	0.568			
10. As a child, I received affective touch from friends/siblings.	0.567			
11. Holding hands*		0.798		
12. Hand around the shoulder*		0.795		
13. Touch forearm or arm of other person to give comfort*		0.731		
14. Hugging*		0.725		
15. Caressing/stroking*		0.723		
16. Massaging*		0.708		
17. While growing up I would reject affective touch (e.g. hugs, caresses) from my parents/caregivers. (R)			0.662	
18. I’ve always I liked to receive comforting bodily contact (e.g. hug) from someone I am close to when distressed.			0.616	
19. I recognize in my personal history the need/ desire to avoid physical affective contact (hugs, caress, arm around shoulder) when I am distressed. (R)			0.611	
20. As a child, I did not like to be hugged by my family members or friends. (R)			0.605	
21. I have always liked to receive caresses from someone that I am close to.			0.514	
22. How frequently you experienced affective touch in different life moments? (Adulthood).				-0.823
23. In my adult life I have given affective touch to close friends or family members.				-0.779
24. In my adult life I have received affective touch from close friends or family members.				-0.679
25. I’ve always I found it easy to comfort friends/family members by hugging or touching their hand/arm.				-0.669
36. How happy you are with the amount of affective touch you experienced in close relationships, in different moments of your life? (Adulthood).				-0.641
27. I recognize in my personal history that I use affective touch (e.g.: hugs, caress, gentle touch in the arm) as a way to communicate affection.				-0.554
28. I am satisfied with the amount of affective touch I gave to others throughout my personal story.				-0.526

Item loadings for 4-component structure of the Tactile Biography on basis of 27 items. Items marked with * were preceded by the question *How comfortable do you feel with these types of affective interpersonal touch in close (romantic and non-romantic) relationships*? Items that were reversed before scoring are marked with (R). Percentages next to component numbers represent the explained variance of the component.

Components were named considering the characteristics of the grouped items: component 1, Childhood/Adolescent Touch Experience (CATE); component 2, Comfort with Interpersonal Touch (CoT); component 3, Fondness for Interpersonal Touch (FoT); component 4, Adult Touch Experience.

Component scores were computed as mean score of the items loading on each component.

### Correlations with Touch Experiences and Attitudes Questionnaire

Table 3 shows Pearson’s correlations between the Tactile Biography (TBIO) components and the subscales of the TEAQ questionnaire. Strong significant correlations were found between the TBIO components and TEAQ factors, suggesting initial good construct validity.

**Table 3:** Correlations TBIO—TEAQ.

	Adult Touch Experience		Childhood/Adolescent Touch Experience		Comfort with Interpersonal Touch		Fondness for Interpersonal Touch	
	r	*p*	R	*p*	r	*p*	R	*p*
**FFT**	.66	.000**	.40	.000**	.52	.000**	.41	.000**
**CIT**	.52	.000**	.37	.000**	.49	.000**	.31	.000**
**ChT**	.39	.000**	.83	.000**	.21	.002**	.35	.000**
**ASC**	.13	.052	.14	.040*	.16	.020*	.12	.074
**AIT**	.40	.000**	.21	.002**	.64	.000**	.36	.000**
**AUT**	.31	.000**	.11	.096	.39	.000**	.28	.000**

Correlation between the component of the Tactile Biography and the Touch experiences and Attitudes Questionnaire. FFT: Friends and Family Touch; CIT: Current Intimate Touch; ChT: childhood Touch; ASC: Attitude to Self-Care; AIT: Attitude to Intimate Touch; Attitude to Unfamiliar Touch.

Significant p values are flagged with * when p < .05 and ** when p < .01.

### Effects of Tactile Biography and attachment styles

#### Regression models

*Earlier touch experiences*, *adult touch experiences and touch deprivation*. Using the continuous component scores from the Tactile Biography *earlier (childhood/adolescent) touch experience* was tested as a predictor for current touch exposure. We found a significant predictive effect to both *touch absence* (p < .001) and *longing* (p = .001) in the regression models. Additionally, and also with the continuous component scores from the Tactile Biography, we tested whether *adult touch experience* was significantly predicted by *earlier (childhood/adolescent) touch experience* or by *style of attachment*. We found that *earlier touch* was also a significant predictor of *adult tactile experiences* (p < .001).

*Attachment styles*, *touch experiences and touch deprivation*. To test whether attachment styles could predict current touch experiences and exposure, the adult touch experience component of the TBIO and subscales of the TDS were separately included as outcomes in a regression model. Adult touch experience, in terms of frequency of and satisfaction with touch, was associated with *attachment avoidance* (p < .001) when individual differences in attachment styles were tested as predictors in the model. In addition, both attachment avoidance and anxiety were significant predictors of *absence of touch* (*p* value < .01 and *p* value < .001 respectively). However, only attachment anxiety predicted *longing for touch* (*p* < .001).

Finally, we tested whether *earlier touch experiences* would predict adult *attachment styles*. Results of the regression models showed that only *attachment avoidance* (p < .001) was significantly predicted by *childhood/adolescent touch experience*.

A complete table with regression coefficients and significance values for each performed regression can be found in supplementary materials.

Taken into consideration the diversity in the composition of the sample, we performed a second regression analysis to determine whether the significant effects found in our regression models were due to confounding effects of differences in sample characteristics. Age and Country of Origin were then added as covariates in all models. All initial results remained significant, suggesting that the effects found were not only attributable to these baseline differences.

Taken together, these results suggest that earlier experiences of affective touch across the lifespan could have a predictive value on adult touch experiences, including the perception of touch deprivation.

According to our expectations, attachment style was found to be associated with the adult touch experiences and with perceived touch deprivation. However, only higher levels of attachment avoidance were associated with lowers rating for both the earlier and the adult touch experience. In terms of touch deprivation, while higher levels of attachment-related anxiety predicted higher levels of both absence of and longing for touch, higher attachment-related avoidance only predicted higher absence of touch but not an increase desire to be touched.

#### Mediation analyses

**Fig 1:**
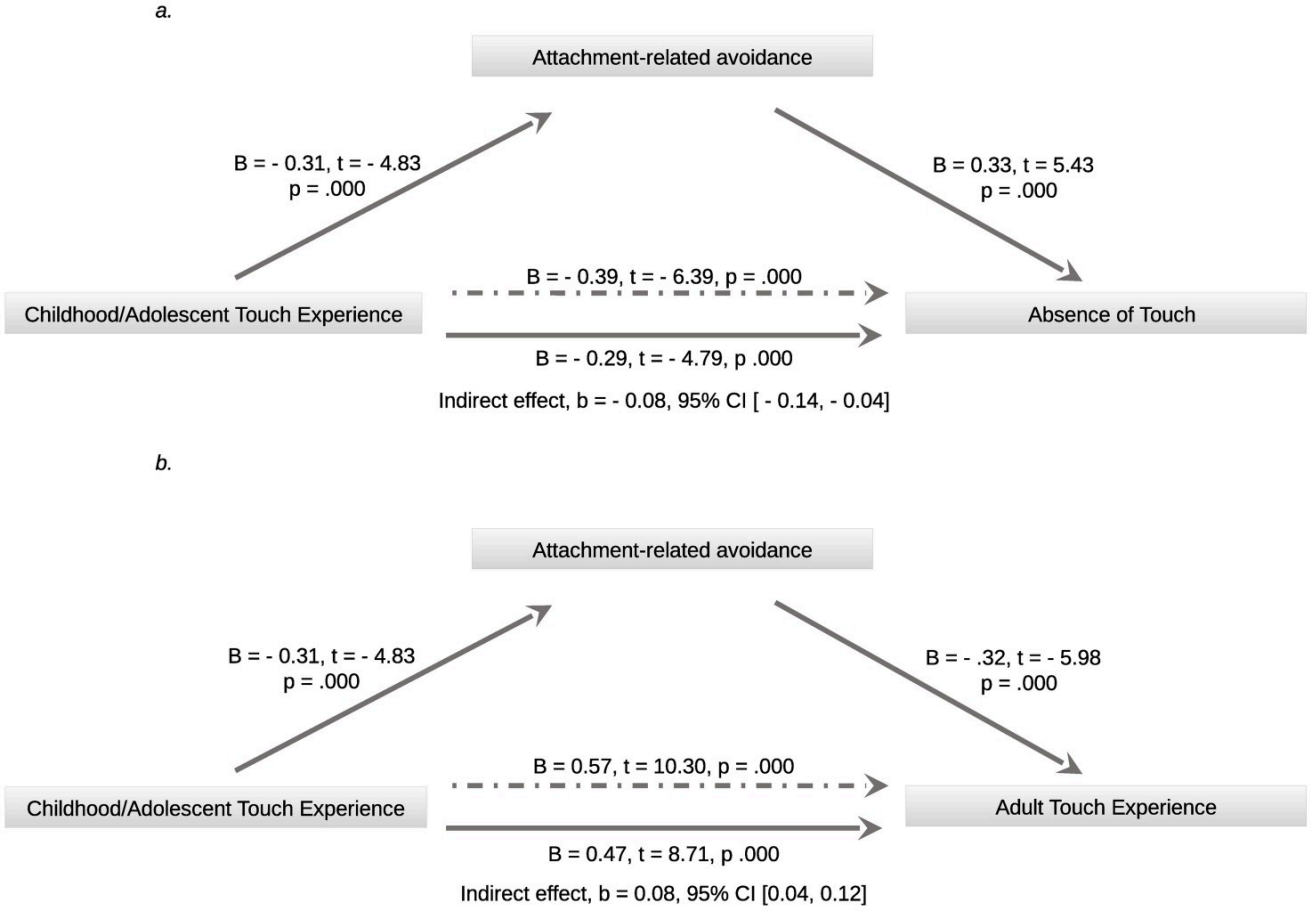
Mediation models. *a*. Model of childhood/adolescent touch as predictor of current touch exposure, mediated by attachment-related avoidance. *b*. Model of childhood/adolescent touch as predictor of adult touch experience, mediated by attachment-related avoidance. Dotted arrows represent total effect of predictor on outcome when other variables are not included in the model. Confidence intervals reported for the indirect effect are bootstrapped based on 5000 samples.

As a second step, mediation analyses were performed among the variables that presented significant regressions. As attachment-related avoidance, but not attachment-related anxiety, was significantly predicted by earlier touch experiences, we only included this variable in the mediation analyses. Two separate mediation models were performed, including both outcomes that were significantly predicted by attachment avoidance: adult touch experiences and perceived touch absence. Results of the models showed that attachment-related avoidance mediated the relationship between childhood/adolescent touch experience and perceived absence of touch (final model: R^2^ = .25, F = 37.73, p = .000), as well as the relationship between childhood/adolescent touch experience and adult touch experience (final model: R^2^ = .41, F = 79.20, p = .000). Fig 1 reports details and significant effects for both models.

In line with our expectations, the indirect effect of attachment style on the relationship between earlier (childhood/adolescent) touch experience and adult touch experience was significant. This also holds true for the relationship between earlier touch and perceived touch deprivation. However, only attachment-related avoidance was found to be a mediator. Attachment-related anxiety, although significantly predicting touch deprivation, was not associated with earlier touch experiences and ergo it was not feasible to consider it as a potential mediator between earlier touch and touch deprivation.

### Negative experiences related to interpersonal touch

Differences in attachment and touch exposure were explored between individuals that reported having a negative experience regarding interpersonal touch during their personal history and those who did not by means of independent samples T-tests. Analysis was performed on 216 participants that responded to the question (yes = 78; no = 138). No significant differences in group composition were found in terms of, age, gender, nationality or family situation.

**Fig 2:**
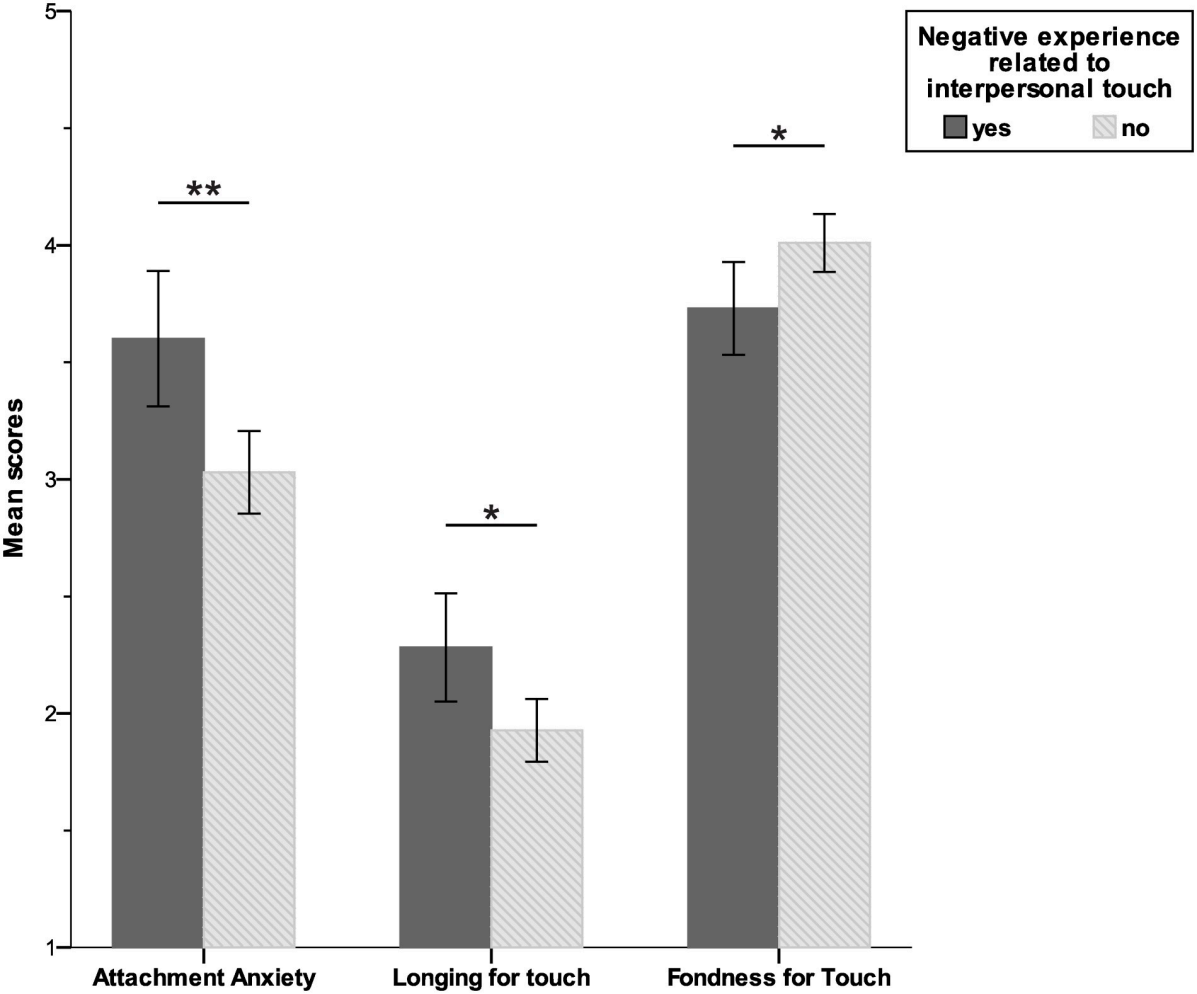
Attachment and touch exposure upon reporting of negative experiences in TBIO. Significant differences in attachment and touch attitudes in participants with and without past negative experiences regarding interpersonal touch. Error bars represent standard error of the mean. * p < .05, ** p < .001.

Participants reporting negative interpersonal touch experiences showed higher levels of attachment related anxiety (M = 3.59 SD = 1.29), than those who did not (M = 3.03 SD = 1.04), t(132.40) = 3.26, *p* = .001; Fig 2. Levene’s test indicated unequal variances (F = 4.69, *p* = .031), so degrees of freedom were adjusted.

Also negative interpersonal touch experiences had a significant impact on the longing for touch subscale of the tactile deprivation questionnaire t(128.75) = 2.48, *p* = .015, with participants that had a negative experience scoring higher (M = 2.26, SD = 1.02) than those who did not (M = 1.93, SD = .80); Fig 2. Again, degrees of freedom were adjusted on basis of Levene’s test (F = 5.67, *p* = .018).

We additionally tested whether there were differences regarding comfort with touch and fondness for touch (as explored by the Tactile Biography) between individuals that had reported a negative experience and those who had not. The fondness for touch component of the TBIO showed significant differences between groups t(213) = -2.32, *p* = .021; Fig 2. In this case participants without self-report of negative experiences showed greater fondness for touch in interpersonal relationships (M = 4.01, SD = .77) in comparison to those who had a negative experience (M = 3.75, SD = .87).

## Discussion

The relationship between tactile interactions across the lifespan and human attachment has been of great interest in many fields of research. This study focused the relation between tactile experiences and human attachment. For this, a Tactile Biography was developed, a new instrument that operationalizes individual differences in the affective touch history across the lifespan. We conducted a set of regression models to explore the relationship between earlier (childhood/adolescence) and later (adulthood) affective touch experiences and exposure, and performed a mediation analysis to examine the role of attachment in this relationship.

After a PCA, we identified a 4-component structure for the Tactile Biography. Two components provided a distinction between earlier (childhood/adolescence) and later (adult) affective touch experiences. These components clustered questions related to both frequency and satisfaction with affective touch. Additionally, two other components were identified related to comfort with interpersonal touch in close relationships and with fondness for interpersonal touch. This 4-component structure seemed to show good internal consistency and each component presented strong correlations with similar subscales from other validated instrument about touch experiences and attitudes (TEAQ). The development of the TBIO adds to the already available validated scales that quantify touch throughout the lifespan. In comparison, the TBIO provides, by means of its diversity in structure (i.e. including emotion identification, negative experiences), a richer idea of the tactile experiences that could be used for descriptive purposes and as part of clinical interviews. The length of the final instrument (35 questions) makes it also appropriate for clinical settings.

The results obtained from the TBIO were subsequently analysed in relation to differences in attachment styles and tactile deprivation. Our results showed that childhood and adolescent touch experiences were significant predictors of individual differences in the style of attachment. Specifically, higher appreciation of earlier touch in terms of frequency and satisfaction, was associated with lower attachment avoidance scores. These results builds on previous research highlighting the close relationship of early tactile interactions for the development of secure attachment [31, 32, 63, 64]. As an example, seminal research from Anisfeld showed that higher frequencies in maternal physical contact with their 3 ½ month old babies led to subsequent increased security of attachment in the dyad [32]. Though not specific to tactile interactions, a longitudinal study showed higher caregiver nurturance at age 3 was associated with declines in attachment avoidance in adolescent in young adults [65]. Recently, Spitoni and collaborators [49] experimentally showed that attachment patterns affect the perception of affective touch in adults. They examined whether people with disorganized attachment (scores that were related to experiences of loss, abuse, or both regarding primary caregivers) perceived affective touch as being less pleasant than those with organized attachment. Their results indicate that adults with a disorganized attachment did not perceive affective touch as pleasant as adults with organized attachment, but rather showed a preference for Non-affective stimulation.

It is important to acknowledge that both earlier touch and adult touch from the TBIO were associated with attachment avoidance but not attachment anxiety. A potential explanation for the link between earlier touch an attachment avoidance in particular could be the suggested association between lower self-reported frequencies of early parental touch with a poorer ‘other’ image, which in turn is potentially indicative of higher attachment related avoidance [42]. In addition, while avoidant individuals can perceive touch as aversive, anxious individuals present more ambivalent feelings towards interpersonal touch [43, 66]. This could explain the lack of significant results between attachment anxiety and the touch experiences dimensions from the TBIO. In line with our results, Chopik et al. [67] showed that while attachment avoidance predicted feelings toward cuddling in romantic and parent–child relationships, attachment anxiety was unrelated to feelings about intimate touch across relationships.

In relation to touch deprivation, we found that attachment related anxiety was a significant predictor of both subscales of the touch deprivation questionnaire that was administered. Higher anxiety was associated with greater perception of absence of touch as well as greater desire to be touched. Conversely, higher attachment avoidance was associated with greater absence of touch but not with the desire to be touched. This is consistent with prior work demonstrating how attachment orientation can influence the preference and interpretation of interpersonal touch (for full review see [27, 44]). As mentioned before, avoidantly attached individuals reportedly hold aversive attitudes towards social touch [45, 67]. Further support comes from experimental touch perception studies. Individuals with self-reported low touch exposure (as would be the case for higher attachment avoidance scores) rated touch as less pleasant than individuals with greater exposure to touch, in particular when it was given at caress type velocities [18]. This could underlie our findings regarding avoidance as a predictor of touch absence but not touch longing of the implemented touch deprivation questionnaire. Conversely, attachment anxiety has been related to the desire of more touch in intimate relationships and to the use of touch for both care-giving and care-seeking reasons [46]. The predictive significance of attachment anxiety for the perception of touch absence and the longing for touch should be contemplated when considering potential sources of bias of touch deprivation. Earlier (childhood adolescence) tactile experience was also found to be a significant predictor of overall appreciation of adult touch experience, in terms of frequency and satisfaction, as measured by the Tactile Biography questionnaire. Moreover, earlier touch experience was a predictor of current perception of touch deprivation. In particular, participants reporting greater scores on the child/adolescent touch component from the Tactile Biography (reflecting higher frequency/satisfaction with touch) showed lower scores on both subscales of the TDS: absence of touch and longing for touch. This suggests that the way in which people process and respond to tactile affective information is intertwined in their personal history of earlier affective touch interactions. Further experimental affective touch research contemplating previous tactile history as a covariate would be a valuable add on to current state of knowledge on the mechanisms modulating touch processing.

Given the diversity of the sample, all regression analyses were adjusted for age and country of origin. Though initial significance was not affected it is important to highlight that touch perception is sensitive to the social and cultural context as well as age [68–71]. The specific impact of these and other variables (such as i.e. instruction level, living conditions) in the relationship between touch and attachment warrants further research.

To the best of our knowledge, no previous studies examined the mediator role of attachment style in the relationship between earlier and later touch. Here, upon finding significant results in the regression models, we explored whether attachment related avoidance was mediating the relationship between earlier and later touch experiences, and also between earlier touch experiences and perceived absence of touch in adulthood. In line with our expectations, we found a mediator role of attachment-related avoidance in both relationships. This result is in line with previous studies linking childhood memories and attachment security. Recollections of family warmth and parental support during childhood have been found to predict life satisfaction in adulthood, with attachment security serving as a mediator of this relationship [72]. This finding could also be of interest for studies of affective touch in psychiatric populations, where patients tend to report an overall low touch frequency regardless of their living situation [73]. Also, experimental studies investigating affective touch in patients undergoing psychotherapy [73], in anorexia nervosa [74] and in adults with autism spectrum disorder [75] have shown that tactile anhedonia (i.e. reduced pleasantness when affectively touched) is present in these populations. These differences in perception and processing of affective touch have not yet been linked to touch frequencies in early life/adulthood. Considering the report from Sailer and Ackerley indicating that exposure to touch can modulate its perception [18], the combination of measures evaluating affective touch perception and the exploring earlier and current experiences of interpersonal touch, might be helpful to further contextualise the findings of tactile anhedonia in psychiatric patients.

Finally, the presence of negative events from the affective tactile history were associated to higher attachment-related anxiety and a decrease in decrease in scores from the TBIO indicating fondness for interpersonal touch. This attitude towards touch is supported by the results of a recent study from Strauss et al. [76]. In an experimental setting, these researchers showed that female patients diagnosed with PTSD (related to physical maltreatment and sexual abuse in their personal stories) perceived touch in diverse conditions as less pleasant and more intense compared to a controls group. In particular, in contrast with controls, patients rated interpersonal stroking touch as negative. Interestingly, although in our study the report of a negative experience led to lower “fondness for interpersonal touch” scores, participants from this group presented a higher desire to be touched. Although this might seem contradictory, it could also relate to the detected increase in anxiety. By exhibiting greater attachment insecurity, these individuals could be prone to seek for reassurance in their close ones through touch, highlighting the relevance of the stress buffer function of social touch [11, 77]. Previous literature has established a link between child abuse and greater attachment insecurity [78]. However, it should be noted that this type of experiences exceeds the mere tactile domain and present multiple and multidimensional aspects for analyses and discussion. It is also important to emphasize, that not all the negative experiences here were necessary reported as childhood trauma. Our question explored the presence of experiences that were perceived as negative, independent of its characteristics. Further research on social touch giving further attention to timing and nature of negatively valued tactile experiences could be enlightening. New studies exploring affective touch perception in particular, could benefit from considering the existence of negative valued touch experiences as a potential modulating variable.

There are limitations present in the current study that need to be mentioned. First, the development of a comprehensive measure than can account for individual differences in the affective tactile history of a person is a challenging task. A tactile affective biography constitutes a rich and complex construct, and as such, efforts to quantify it can be reductionist. Type and quality of interpersonal touch is likely to vary across the spectrum of the adult relationships that are established with others (familial, non-familial, romantic, etc), and in a more exhaustive version of this instrument these differences should also be acknowledged. Also, as a self-reported measure based on recollection of past experiences, caution should be taken when interpreting results. A downside of self- report questionnaires, in which participants are asked to report previous experiences, is the potential lack of accuracy in the recollection of events. In addition to memory and social desirability biases, other types of bias could also affect results. For example, individuals that are more satisfied with present touch interactions could be also prone to have more positive recollections of past experiences. However, while acknowledging that 100% accuracy may not be possible, self-report still constitutes a valuable tool to reconstruct previous experiences in the field of affective touch research. The development of a tactile biography interview, by means of other recollection techniques, could help address this issue and add value to future studies.

Second, although the initial development of the Tactile Biography seems promising, further development and validation of this instrument using confirmatory factor analysis should be performed in a second sample of participants. Furthermore, we acknowledge that attachment constitutes as well a multidimensional and complex construct and further research could benefit from inclusion of other measures of attachment such as the Adult Attachment Interview [79]. Inclusion this type of measures could be of particular relevance when analysing attachment and self-reported measures of social tactile interactions, considering potential social desirability biases and memory biases of retrospective reporting. For example, a recent study suggests that individuals with high levels of attachment anxiety tend to experience inaccurate memories [80].

Third, education level was high across our sample, with >90% of participants with post-secondary studies and >50% with university studies. This can be limiting factor for the generalizability of the study results; further research would benefit from more diversity in the study sample regarding level of education.

In addition, the Touch Deprivation Scale is an instrument that so far has been only validated on a sample of university students. Considering the diverse composition of the sample age (range 18 to 81 years—average 37) there may be fundamental age-related differences regarding deprivation of touch, in particular in relation to sexuality. The low reliability value of this subscale (with the consequent exclusion form the analysis) could be due partly due to this issue.

Lastly, it is important to acknowledge that the interpretation of the association between childhood and adolescent touch experiences and individual differences in the attachment style requires additional evidence. Spurious associations could be caused by a large variety of contextual factors and interpersonal variables (i.e. genetics; [81, 82]) that might be acting as confounders to both touch experiences and attachment. Here it should be considered that, though relatively stable, attachment is dynamic and presents fluctuations across the lifespan. [24, 70, 83]. Although recent longitudinal studies provide further evidence of an association between early caregiving experiences and adult attachment styles (i.e. [65, 81]), Fraley and Roisman argue that these associations are relatively small and there is no consistent set of predictors based on early experiences for later outcomes [84]. In addition, these authors suggest that although attachment styles are relatively more malleable earlier in life (due to socialization- selection asymmetries), early experiences are not deterministic and attachment developmental trajectories are shaped by multiple and potentially competing experiences throughout life (see also [85]). In this sense and as previously mentioned, attachment styles are updated in every relational interaction across the lifespan [24]. On-going attachment experiences are important for understanding interpersonal functioning and can provide a better insight on attachment that earlier experiences alone [84].

Nevertheless, the Tactile Biography we developed appears to be a useful instrument to approach individual differences in affective tactile experiences in the context of close relationships and across the lifespan. Results from the Tactile Biography showed that interpersonal affective touch experiences earlier in life may affect how we experience affective touch in the long-term. Moreover, our results suggest a possible model supporting the role of attachment as a mediator between childhood/adolescent affective tactile experiences and tactile deprivation in adulthood. Longitudinal studies, contemplating quantitative and qualitative features of tactile interactions and attachment dynamics throughout the lifespan, could be a valuable focus for further research.

## Conclusions

The current study developed the Tactile Biography which assesses individual differences in affective touch experiences across the lifespan. Additionally, results of the regression analyses performed suggest that reported experiences of affective touch during childhood and adolescence seem to be closely associated with adult attachment styles and adult social touch experiences. Avoidant attachment appears to serve as a mediator in the relationship between earlier (childhood/adolescent) and later (adult) affective touch experiences, as well as between earlier (childhood/adolescent) affective touch experiences and perceived touch deprivation in adulthood.

These findings offer further support to existing literature highlighting the relevance of touch and attachment throughout life, and provide novel insights for the fields of social touch and attachment research.

## Supporting information

S1 Appendix Participants data. General characteristics and questionnaire scores.
(XLSX)

S2 Appendix Tactile Biography. (PDF)

S3 Appendix Regression table. (DOCX)
