# Supplementary material for: Affective touch experiences across the lifespan: Development of the Tactile Biography questionnaire and the mediating role of attachment style
Source: PLoS One. 2020 Oct 28;15(10):e0241041. doi: 10.1371/journal.pone.0241041 (PMC7592771; doi:10.1371/journal.pone.0241041)
Supplement: S2 Appendix — (PDF) [file pone.0241041.s002.pdf]

S2. Appendix. Tactile Biography

(Beltrán, Dijkerman, Keizer)

The following questions refer to experiences of affective touch in close relationships throughout your personal history. Affective touch refers in this questionnaire to activities where people touch each other affectionately, such as embracing, holding hands, cuddling, stroking, caressing, putting a hand around other's shoulder, etc.

Please select a response for each question according to the highlighted indication on top of each section.

**Please select a response next to each of the statements below to indicate how frequent the stated event occurred.**<br>1= Never – I cannot recollect event ever occurring<br>2 = Rarely (less than once a week)<br>3 = Occasionally (once a week or more)<br>4 = Frequently (every day)<br>5 = Very frequently (more than once a day)			
1.	As a child I received affective touch from family members (parents/caregivers).	1 Never	5 Very frequently
2.	As a child my parents/caregivers would use bodily contact (e.g.: caressing, hugging, etc) to comfort me when ill/distressed.	1 Never	5 Very frequently
3.	As a child my parents/caregivers would use bodily contact (e.g.: caressing, hugging, etc) to congratulate me/ give me positive feedback.	1 Never	5 Very frequently
4.	As a child, I received affective touch from friends/siblings.	1 Never	5 Very frequently
5.	In my adult life I have received affective touch from close friends or family members	1 Never	5 Very frequently
6.	In my adult life I have given affective touch to close friends or family members	1 Never	5 Very frequently
	**Please indicate how frequently you experienced affective touch in these different life moments:**		
7.	a. Childhood	1 Never	5 Very frequently
8.	b. Adolescence	1 Never	5 Very frequently
9.	c. Adulthood	1 Never	5 Very frequently
**Please select a response for each of the statements below to indicate the degree to which each statement applies to you.**			
10.	As a child, I did not like to be hugged by my family members or friends.	1 Not at all true	5 Extremely true
11.	I have always liked to receive caresses from someone that I am close to.	1 Not at all true	5 Extremely true
12.	I've always I liked to receive comforting bodily contact (e.g. hug) from someone I am close to when distressed	1 Not at all true	5 Extremely true
13.	I've always I found it easy to comfort friends/family members by hugging or touching their hand/arm	1 Not at all true	5 Extremely true
14.	I recognize in my personal history that I use affective touch (e.g.: hugs, caress, gentle touch in the arm) as a way to communicate affection.	1 Not at all true	5 Extremely true
15.	While growing up, upon stressful situations I would go to my parents/caregivers in search of affective touch (hugs, cuddling, caressing)	1 Not at all true	5 Extremely true
16.	While growing up I would reject affective touch (e.g. hugs, caresses) from my parents/caregivers.	1 Not at all true	5 Extremely true
17.	I recognize in my personal history the need/desire of physical affective contact (hugs, caress, arm around shoulder) when I am distressed.	1 Not at all true	5 Extremely true

18.	I recognize in my personal history the need/ desire to avoid physical affective contact (hugs, caress, arm around shoulder) when I am distressed.	1 Not at all true — 5 Extremely true
19.	I am satisfied with the amount of affective touch I received throughout my personal story.	1 Not at all true — 5 Extremely true
20.	I am satisfied with the amount of affective touch I gave to others throughout my personal story.	1 Not at all true — 5 Extremely true
	**Please indicate how comfortable do you feel with these types of affective interpersonal touch in close (romantic and non-romantic) relationships**	
21.	a. Holding hands	1 Very uncomfortable — 5 Very comfortable
22.	b. Hand around the shoulder	1 Very uncomfortable — 5 Very comfortable
23.	c. Touch forearm or arm of other person to give comfort	1 Very uncomfortable — 5 Very comfortable
24.	d. Hugging	1 Very uncomfortable — 5 Very comfortable
25.	e. Caressing/stroking	1 Very uncomfortable — 5 Very comfortable
26.	f. Massaging	1 Very uncomfortable — 5 Very comfortable
	**How happy you are with the amount of affective touch you experienced in close relationships, in different moments of your life?**	
27.	a. Childhood	1 Not at all happy — 5 Extremely happy
28.	b. Adolescence	1 Not at all happy — 5 Extremely happy
29.	c. Adulthood	1 Not at all happy — 5 Extremely happy

30. Affective touch in close interpersonal relationships generates in me… (selecting more than 1 option is valid)

- ☐ Calmness/relaxation
- ☐ Embarrassmentt
- ☐ Enjoyment
- ☐ Happiness
- ☐ Rejection
- ☐ Disgust
- ☐ Comfort
- ☐ Irritation
- ☐ Discomfort
- ☐ Pleasure

31. I recognize in my personal history the presence of negative/unpleasant experiences involving interpersonal touch.

- ☐ Yes
- ☐ No
- ☐ Prefer Not to Answer

If yes: Could you name what type of experience? ....

32. In close relationships have always preferred to:

- ☐ give affective touch
- ☐ receive affective touch
- ☐ does not matter (I like both the same)
- ☐ does not matter (I dislike both the same)

** Items marked with (R) are reversed scored.*
