# Supplementary material for: Affective touch experiences across the lifespan: Development of the Tactile Biography questionnaire and the mediating role of attachment style
Source: PLoS One. 2020 Oct 28;15(10):e0241041. doi: 10.1371/journal.pone.0241041 (PMC7592771; doi:10.1371/journal.pone.0241041)
Supplement: S3 Appendix — (DOCX) [file pone.0241041.s003.docx]

**S3. Appendix. Regression table.**

Regression models. Names of subscales are followed by indication of the main questionnaire to which they belong. ECR-R = Experiences in Close Relationships -Revised. TDS= Touch Deprivation Scale. TBIO= Tactile Biography. Ch/Ad Touch: Childhood and Adolescence Touch Experience from Tactile Biography. *Country represents a dummy variable built as country majority (in %) vs other countries. Coefficient values are followed by bootstrapped 95% CI indicated between brackets.

a.

| Outcome Variable | | Absence of Touch  (subscale TDS) | | | | Longing for Touch  (subscale TDS) | | | | Adult Touch Experience  (subscale TBIO) | | | |
| --- | --- | --- | --- | --- | --- | --- | --- | --- | --- | --- | --- | --- | --- |
|  | | b | SE | B | p | b | SE | B | p | b | SE | B | p |
| STEP 1 | (Constant) | .68  (.27 , 1.08) | 0.22 |  | .004 | 1.12  (.63, 1.60) | 0.25 |  | .000 | 5.42  (5.10 , 5.73) | 0.16 |  | .001 |
|  | ECR Avoidance | .32  (.19 , .44) | 0.06 | .37 | .001 | 0.02  (- .12, .16) | 0.07 | .02 | .792 | -.37  (-.47 , -.27) | 0.05 | -.44 | .001 |
|  | ECR Anxiety | .11  (.03 , .19) | 0.04 | .16 | .010 | 0.27  (.18, .37) | 0.05 | .36 | .001 | -.05  (-.12 , .04) | 0.04 | -.08 | .268 |
| STEP 2 | (Constant) | .37  (-.11 , .84) | 0.24 |  | .125 | .902  (.28, 1.50) | 0.30 |  | .002 | 5.25  (4.91 , 5.63) | 0.20 |  | .001 |
|  | ECR Avoidance | .30  (.17 , .42) | 0.06 | .35 | .001 | .006  (- .14, .15) | 0.07 | .01 | .941 | -.36  (-.45 , -.26) | 0.05 | -.42 | .001 |
|  | ECR Anxiety | .13  (.05 , .21) | 0.04 | .20 | .003 | 0.29  (.19, .39) | 0.05 | .37 | .001 | -.07  (-.14 , .01) | 0.04 | -.11 | .084 |
|  | Age (in years) | .01  (-.001 , .01) | 0.00 | .11 | .110 | .004  (- .004, .12) | 0.00 | .06 | .317 | .008  (.00 , .01) | 0.00 | .14 | .008 |
|  | Country * | .25  (.07 , .45) | 0.09 | .17 | .011 | .15  (- .82, .41) | 0.12 | .08 | .212 | -.25  (-.43 , -.08) | 0.08 | -.17 | .003 |

b.

| Outcome Variable | | Absence of Touch  (subscale TDS) | | | | Longing for Touch  (subscale TDS) | | | | Adult Touch Experience  (subscale TBIO) | | | |
| --- | --- | --- | --- | --- | --- | --- | --- | --- | --- | --- | --- | --- | --- |
|  | | b | SE | *B* | *p* | b | SE | B | *p* | b | SE | B | *p* |
| STEP 1 | (Constant) | 3.33  (2.94, 3.77) | 0.21 |  | .001 | 2.88  (2.36, 3.39) | 0.26 |  | .001 | 2.32  (1.88, 2.74) | 0.22 |  | .001 |
|  | Ch/Ad Touch | - .33  (-.44, - 22) | 0.05 | -.40 | .001 | - .22  (-.35, -.08) | 0.07 | -.22 | .002 | .46  (.35, .57) | 0.06 | .57 | .001 |
| STEP 2 | (Constant) | 3.02  (2.52, 3.56) | 0.25 |  | .001 | 2.75  (2.04, 3.40) | 0.32 |  | .001 | 2.09  (1.63, 2.62) | 0.25 |  | .001 |
|  | Ch/Ad Touch | - .32  (-.42, -.21) | 0.50 | -.38 | .001 | - .22  (-.34, -.08) | 0.07 | -.22 | .003 | .45  (.34, .56) | 0.05 | .56 | .001 |
|  | Age (in years) | .01  (-.00, .01) | 0.00 | .09 | .142 | .00  (-.01, .01) | 0.00 | .05 | .503 | .01  (.00, .01) | 0.00 | .16 | .003 |
|  | Country * | .20  (.03, .40) | 0.10 | .13 | .044 | .02  (-.21, .25) | 0.12 | .01 | .864 | - .21  (-.39, -.05) | 0.09 | -.14 | .012 |

c.

| Outcome Variable | | Attachment Anxiety (ECR) | | | | Attachment Avoidance (ECR) | | | |
| --- | --- | --- | --- | --- | --- | --- | --- | --- | --- |
|  | | b | SE | B | p | b | SE | B | p |
| STEP 1 | (Constant) | 3.69  (3.07, 4.33) | 0.33 |  | .001 | 4.44  (3.97, 4.93) | 0.25 |  | .001 |
|  | Ch/Ad Touch | - .12  (-.31, .06) | 0.09 | -.09 | .183 | -0.29  (-0.41, -0.17) | 0.06 | -.31 | .001 |
| STEP 2 | (Constant) | 3.94  (3.18, 4.74) | 0.40 |  | .001 | 4.35  (3.71, 4.97) | 0.32 |  | .001 |
|  | Ch/Ad Touch | -0.13  (-.32, .06) | 0.09 | -.10 | .162 | -.28  (-.42, -.16) | 0.06 | -.30 | .001 |
|  | Age (in years) | -.002  (-.01, .01) | 0.01 | -.02 | .712 | .001  (-.01, .01) | 0.04 | .02 | .810 |
|  | Country * | -.39  (-.67, -.09) | 0.15 | -.16 | . 015 | .08  (-.14, .30) | 0.11 | .05 | .498 |
